# Deep Learning-Based Prediction Model for the Cobb Angle in Adolescent Idiopathic Scoliosis Patients

**DOI:** 10.3390/diagnostics14121263

**Published:** 2024-06-14

**Authors:** Chun-Sing (Elvis) Chui, Zhong He, Tsz-Ping Lam, Ka-Kwan (Kyle) Mak, Hin-Ting (Randy) Ng, Chun-Hai (Ericsson) Fung, Mei-Shuen Chan, Sheung-Wai Law, Yuk-Wai (Wayne) Lee, Lik-Hang (Alec) Hung, Chiu-Wing (Winnie) Chu, Sze-Yi (Sibyl) Mak, Wing-Fung (Edmond) Yau, Zhen Liu, Wu-Jun Li, Zezhang Zhu, Man Yeung (Ronald) Wong, Chun-Yiu (Jack) Cheng, Yong Qiu, Shu-Hang (Patrick) Yung

**Affiliations:** 1 Department of Orthopaedics and Traumatology, The Chinese University of Hong Kong, Hong Kong, Chinatplam@cuhk.edu.hk (T.-P.L.); mschan@cuhk.edu.hk (M.-S.C.); lawsw@cuhk.edu.hk (S.-W.L.);; 2 Division of Spine Surgery, Department of Orthopedic Surgery, The Affiliated Hospital of Nanjing University Medical School, Nanjing 210000, China; hezhong_nju@126.com (Z.H.); drliuzhen@163.com (Z.L.); zhuzezhang@126.com (Z.Z.); 3 Department of Orthopaedics and Traumatology, Prince of Wales Hospital, Hong Kong, China; aleclhhung@cuhk.edu.hk; 4 Department of Imaging and Interventional Radiology, The Chinese University of Hong Kong, Hong Kong, China; winniechu@cuhk.edu.hk; 5 Department of Mechanical Engineering, The University of Hong Kong, Hong Kong, China; smak@hku.hk; 6 Koln 3D Technology (Medical) Limited Company, Hong Kong, China; edmond@koln3d-tech.com; 7 National Institute of Healthcare Data Science, Nanjing University, Nanjing 210023, China; liwujun@nju.edu.cn; 8 National Key Laboratory for Novel Software Technology, Department of Computer Science and Technology, Nanjing University, Nanjing 210023, China

**Keywords:** adolescent idiopathic scoliosis (AIS), Cobb angles, Feedforward Neural Network (FNN)

## Abstract

Scoliosis, characterized by spine deformity, is most common in adolescent idiopathic scoliosis (AIS). Manual Cobb angle measurement limitations underscore the need for automated tools. This study employed a vertebral landmark extraction method and Feedforward Neural Network (FNN) to predict scoliosis progression in 79 AIS patients. The novel intervertebral angles matrix format showcased results. The mean absolute error for the intervertebral angle progression was 1.5 degrees, while the Pearson correlation of the predicted Cobb angles was 0.86. The accuracy in classifying Cobb angles (<15°, 15–25°, 25–35°, 35–45°, >45°) was 0.85, with 0.65 sensitivity and 0.91 specificity. The FNN demonstrated superior accuracy, sensitivity, and specificity, aiding in tailored treatments for potential scoliosis progression. Addressing FNNs’ over-fitting issue through strategies like “dropout” or regularization could further enhance their performance. This study presents a promising step towards automated scoliosis diagnosis and prognosis.

## 1. Introduction

Scoliosis is a three-dimensional (3D) structural deformity of the spine, and adolescent idiopathic scoliosis (AIS) is the most common type of scoliosis, affecting around 1–3% of adolescents [1]. Idiopathic indicates that the disease has a little known etiology compared to other types of scoliosis with more clear underlying mechanisms such as neuromuscular or degenerative types.

The gold standard of measuring the curvature of the spine and diagnosing AIS is through the Cobb angle, which is the angle formed by the two most tilted vertebra, the top vertebra and the bottom vertebra, forming the curve based on a standard posteroanterior (PA) radiograph. Scoliosis is clinically defined as when the Cobb angle is ≥10° [2]. The Cobb angle remains the gold standard of diagnosing, monitoring, and managing AIS patients, yet the measurement of the Cobb angle has limitations and is intrinsically complicated [3]. Besides the natural diurnal variation in the Cobb angle, which was found to be up to five degrees [4], or the variations in vertebral rotations due to positioning and radiographs acquisition [5], the intra-observer and inter-observer variation based on the same set of radiographs could contribute to around five degrees and seven degrees of variation [6]. Therefore, there is a need for a tool that is fast and can calculate standardized Cobb angles automatically.

Previous studies have developed an automated system for screening for AIS using a combined algorithm of Faster-RCNN and Res-Net to process unclothed back images. Their results show that the algorithm has better accuracy than experienced clinicians in detecting cases of scoliosis and a curve severity of 20 degrees or more. This automated screening method has the potential to be used as a routine tool for scoliosis detection and disease progression monitoring without radiation exposure to patients. However, cases identified through this method may have mixed etiologies, and further improvements to the accuracy of the algorithm are necessary [7]. Zhang et al. established an automated classification system that can be constructed using radiographs from AIS patients and associated Cobb angle measurements. However, the test set used in these previous studies had a relatively small number of images [8]. The common tools used in AIS screening are the Adam Forward Bending Test, scoliometer, and Moire topography. It has always been a critical task not only to identify AIS patients through screening but also to identify those whose scoliosis will progress to provide continuous radiographical monitoring and non-operative early intervention. Progression factors equations were developed by, for example, Lonstein and Carlson [9] and Yamauchi [10], Soucacos et al. [11], and Zhang et al. [12], with the following factors that appear to be associated with a high risk of curve progression: curve pattern, curve magnitude, chronological age, gender, and maturity as determined by menarche or the Risser sign. Weinstein (1999) [13] summarized a dichotomy classifying the factors as “curve-related” and “maturity-related”, until Hung et al. put forward a new prognostic factor as a third category in 2005 [14], the “bone-related” factor, which was validated in a study in 2013 using quantitative ultrasound (QUS) with 294 AIS girls.

Subsequently, an attempt has been made to reconstruct a 3D spinal model from plain X-ray images, followed by a prediction model of the progression of the spinal curve in a 3D shape [15]. Although the results looked promising, the 3D reconstruction process, as of our best knowledge today, requires trained manpower to undergo a tedious process to identify landmarks in each vertebra [16]. A fully automated scoliosis progression prediction model is still in need. The rise of Artificial Intelligence (AI) in recent years seems to be making this hope possible. In a recent study, a deep learning model was first implemented to predict whether an AIS spine would progress or not progress, based on standing PA X-rays at the first clinic visit [17].

The current study aimed to put this binary prediction (progress or not) model forward. A vertebra landmark extract method [18] for first extracting the intervertebral angles from PA view X-rays with the chest was applied. Next, they were presented in a novel way: A matrix showing all the intervertebral angles from the twelve thoracic vertebrae (T1–T12) to the five lumbar vertebrae (L1–L5). The Cobb angle was extracted by identifying the largest vertebral pair angle. An FNN, an early and simple type of neural network, was further applied to predict the progression of the Cobb angle. A fast and standardized way to identify and present an inter-vertebral angle matrix from an X-ray was demonstrated with the aid of AI. The system provided a further detailed, accurate, and flexible curve progression prediction tool for surgeons to obtain the curve progression information of their concern for treatment and management planning.

## 2. Materials and Methods

### 2.1. Data Collection

The data of the subjects of the current study were retrospectively collected from Nanjing Drum Tower Hospital, China. The inclusion criteria were patients (1) aged 10–18, (2) diagnosed with AIS, (3) with at least three consecutive visits to a hospital in a 4–6-month interval to undergo X-ray examination, (4) with clear X-ray images from T1 to L5 and (5) who underwent no other treatment during the period. In the case of patients with multiple curves, the analysis focused solely on the major curve, which is determined by the largest Cobb angle.

### 2.2. General Workflow and Data Preprocessing

**Figure 1:**
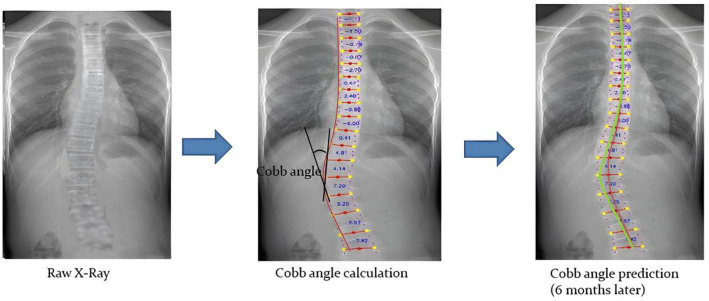
The general workflow of the deep learning model.

The entire workflow of the deep learning model from the input of X-ray images to the calculation of Cobb angles and the prediction of Cobb angles will be briefly and plainly described in Figure 1. The raw DICOM files were converted to JPG format and cropped to contain seventeen spinal vertebrae T1–L5 and the chest.

### 2.3. Vertebra-Focused Landmark Extraction Method

**Figure 2:**
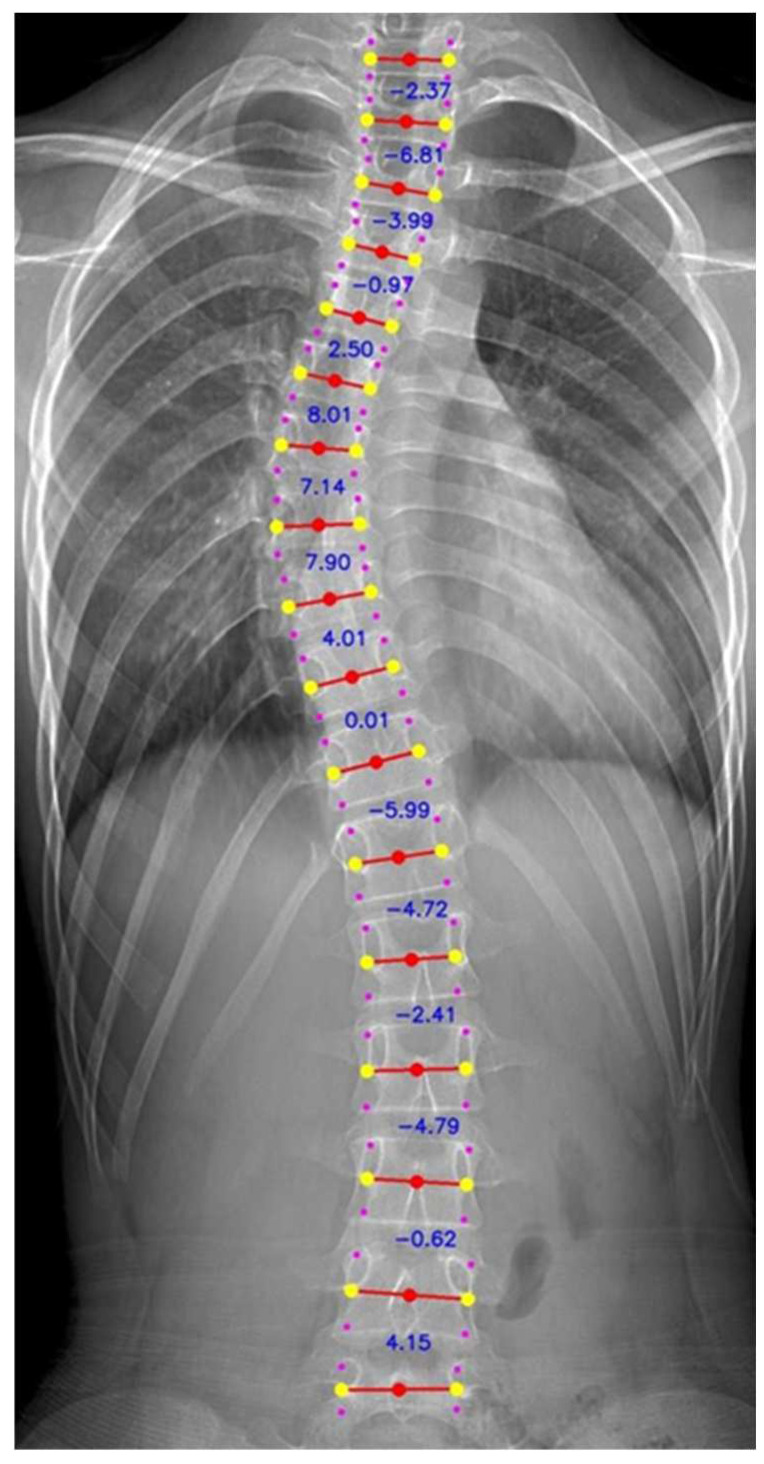
X ray cropped to T1–L5 with the chest and vertebral landmark extracted.

The vertebra extraction method proposed by Yi et al. in 2020 [18] was then applied to locate the landmarks of each vertebra for intervertebral angles calculation (as indicated in Figure 2). The data were then screened manually, and data points with wrongly extracted labels were discarded. The vertebra-focused landmark extraction method is a novel technique in scoliosis assessment that first localizes the center of each vertebra and then traces the four corner landmarks [18]. Compared to the previous regression-based or segmentation-based approach, the method demonstrated a better performance regarding Cobb angle calculation in terms of the symmetric mean absolute percentage error (SMAPE) and vertebra landmark detection in terms of the average detection error. As the center points would not be overlapping with each other, it would not suffer from the touching problems from the segmentation method. The method was also shown to be robust in low-contrast X-ray images.

### 2.4. Cobb Angle and Intervertebral Angles Matrix Calculations

**Figure 3:**
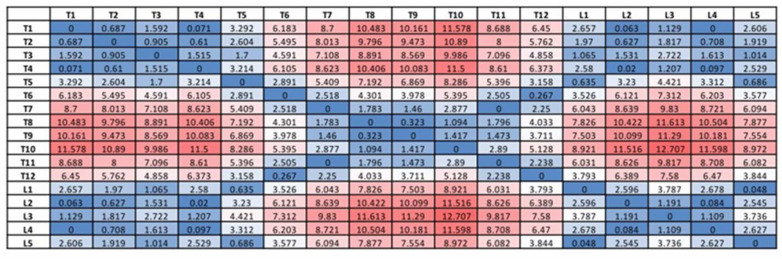
A sample matrix that showcases Cobb angle values between individual vertebral column pairs (e.g., the T1–T2 pair, where T1 is measured against T2 to obtain the Cobb angle between the T1 and T2 vertebral columns). The severity of scoliosis is correlated with the color intensity (more severe scoliosis can be observed as the color approaches dark red).

A novel method in presenting all the intervertebral angles in a matrix format was adopted (as depicted in Figure 3). The Cobb angle was extracted from the intervertebral pair with the highest degree of the angle. The table was presented in a heatmap format, with the higher degree angle showing red and the lower degree showing blue.

### 2.5. FNN and Intervertebral Angles Progression Prediction

**Figure 4:**
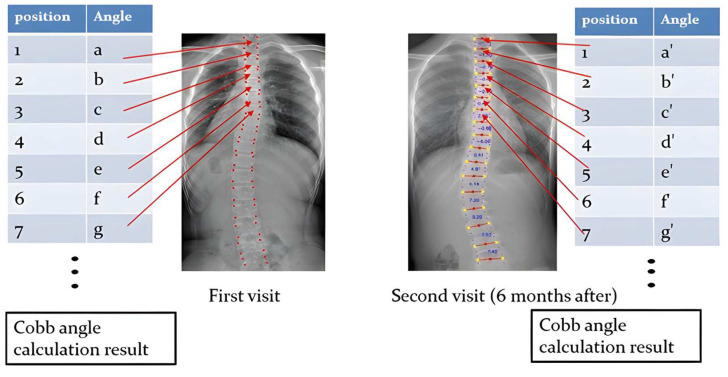
A pair of training data where the first-visit spinal data are the input and the second-visit spinal data are the desirable output for the supervised training of the deep learning model.

**Figure 5:**
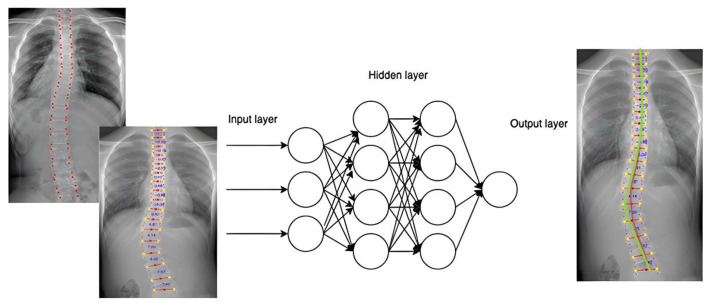
The FNN was at first trained with input data (X-ray images taken during the first visit) and the corresponding X-ray images taken during the consecutive visits (i.e., the second and third visits) as the desirable output. The trained neural network would predict the Cobb angle in the second and third visits (on the **right**) based on the new X-ray image taken during the first visit (on the **left**).

An early and simple yet powerful FNN in predicting the Cobb angle progression was applied. Forward neural networks are commonly used for handling regression as well as classification problems [19]. An FNN is an artificial neural network structured in sequential layers, and each of the nodes is formed by artificial neurons [20]. FNNs are characterized by the fact that the nodes in each layer are not related to each other. It began with an input layer that received input data to be processed (as indicated in Figure 4). The data were then forwarded to one or more hidden layers where the classification/regression happens, and it was finally transferred to the output layer showing the result (as shown in Figure 4). During the process, the backward propagation algorithm helped to improve the prediction model. It is a supervised learning technique for multilayer neural networks. The principle of the backpropagation method is to modify the internal weighting of each input to produce a desired output. For a given input, a desired output was provided as a reference (as illustrated in Figure 5). After providing the input signal, the generated result was compared to the desired result, and a network error was generated. If the classification was not correct, the weights of the neurons would be updated. The process was repeated, and the model should converge to a minimum error.

**Figure 6:**
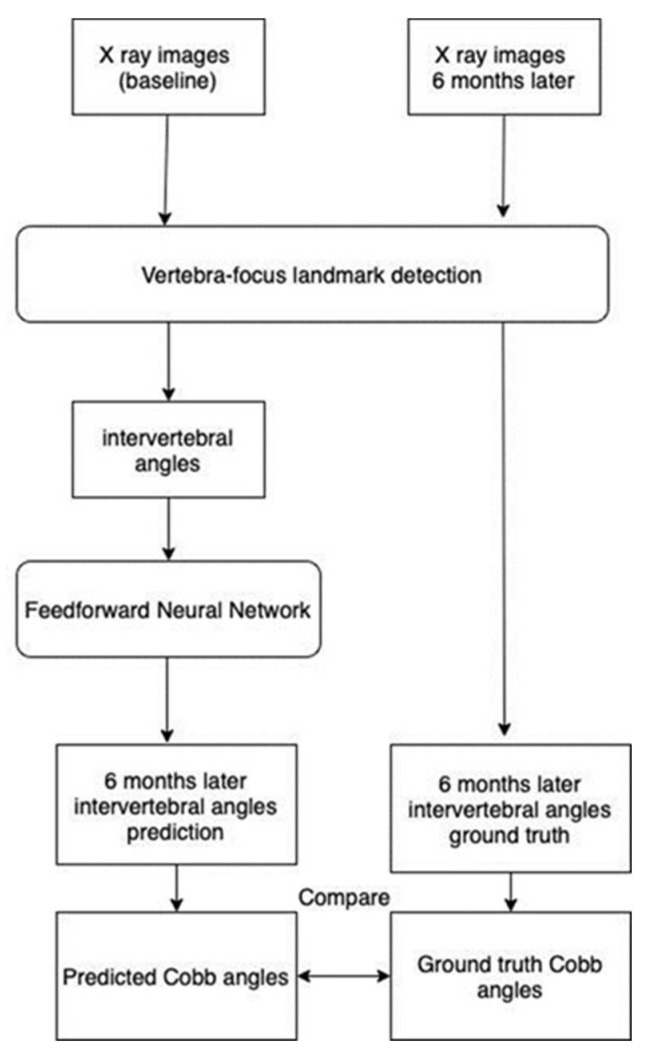
Workflow for the comparison of predicted Cobb angles and ground truth Cobb angles.

A total of 16 basic pairs of intervertebral angles (T1–T2, T2–T3, … L4–L5) for each X-ray image extracted by the vertebra-focus landmark detection method were used to predict the corresponding pair of intervertebral angles 6 months later. The Cobb angle was calculated based on the largest angle formed by the two pair of intervertebral angles. Therefore, a predicted set of intervertebral angles and the Cobb angle were generated. On the other hand, the X-ray images 6 months later were extracted from the intervertebral angles, and the Cobb angle was calculated. These angles were considered as the ground truth. The predicted Cobb angle was used to compare with the ground truth Cobb angle (as displayed in Figure 6). The final model would carry out the Cobb angle calculation based on the input data and perform the angle prediction.

### 2.6. K-Fold Cross-Validation

K-fold cross-validation, a popular model validation technique [21], was adopted in the design. The dataset was randomly divided into a certain number (k) of equal-sized subsets, and k-1 subsets were used for training the model, while the remaining subset was unseen data used for validating the model. The procedure was repeated k times so that k number of performance estimates for each iteration was acquired. Ultimately, the mean of the k number of performance estimates was obtained. In the current design, k was assigned to be five such that the dataset was split into five subsets

### 2.7. Statistical Analysis

To demonstrate the performance of the model, the confusion matrices were calculated after training, providing the values for true positives (TP), true negatives (TN), false positives (FP), and false negatives (FN). The results were considered statistically significant when *p* < 0.05.

## 3. Results

### 3.1. Demographics

There are, in total, 79 patients included in the current study, after 14 patients whose X-ray images could not allow for the correct extraction the intervertebral angles were excluded. There are 67 females and 12 males. The average number of clinic visits was 5.48 (range 3–9), giving us a total of 433 data points for the analysis. The average age at the first clinic visits where the first X ray images were taken was 16.5 years old (range 13–18). The mean Cobb angle at the first clinic visit for the 79 patients was 24.8 degrees (range 12.9–42.6 degree). The intervertebral angle matrix enabled us to observe the Cobb angle, which was constructed from pairs of vertebrae from T1 to L5. At the first clinic visit, there were 36 patients whose Cobb angle was calculated from T1 to T12 (Thoracic), 39 whose Cobb angle was across the thoracic and lumbar regions (Thoracolumbar), and 4 whose Cobb angle was calculated from L1 to L5 (Lumbar).

### 3.2. Intervertebral Angles Matrix

The intervertebral angles were extracted by the vertebra-focused landmark extraction method, and an example is presented in a matrix format (shown in Figure 2). The matrix described a complete picture of the spinal curve of the patients, indicating not only the Cobb angle but also the two vertebrae from which the Cobb angle originated. The spectrum of color indicates the degree of severity of the degree of the angle, from light blue, the smallest angle, to dark red, the largest angle.

### 3.3. Intervertebral Angles Progression Prediction

**Figure 7:**
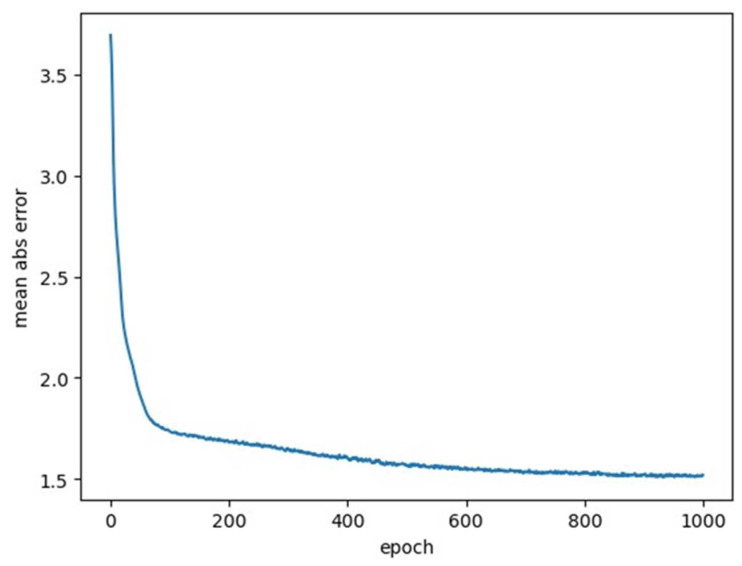
Loss function curve during neural network training (the MAE was 1.5 degrees).

**Figure 8:**
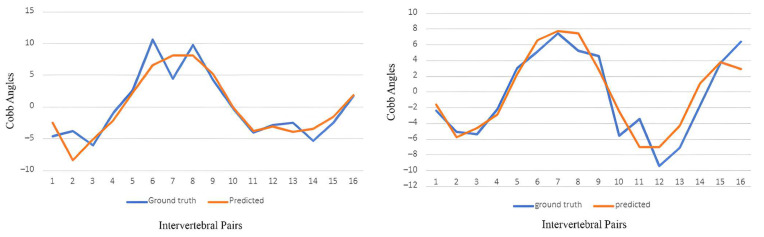
Samples of the predicted and ground truth curve for 16 basic pairs of intervertebral angles.

The prediction of the intervertebral angles progression was based on the 16 basic pairs of intervertebral angles. The predicted angles of these 16 pair angles were compared with the corresponding ground truth pair angles. The loss curve of the machine learning model is presented in Figure 7, and the overall mean absolute error was 1.5 degrees. A sample of the predicted curve over the ground truth curve for the 16 basic pairs of intervertebral angles is presented in Figure 8 below, and the overall overlapping area is high.

### 3.4. Cobb Angle Progression Prediction

**Figure 9:**
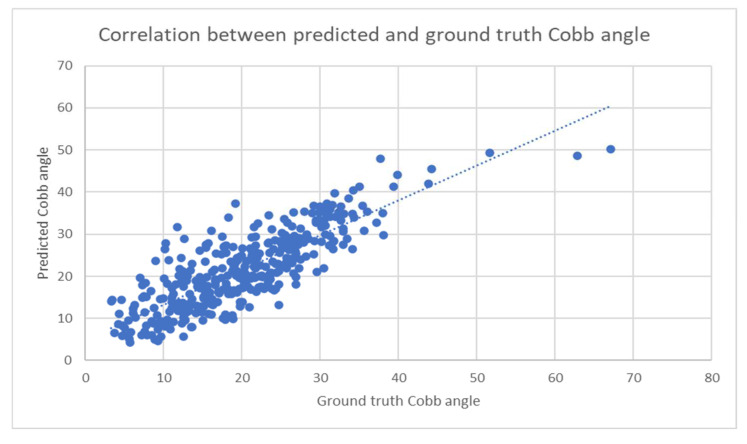
Correlation between predicted and ground truth Cobb angle (Pearson correlation of predicted Cobb angles was 0.86).

Based on the X-ray image and Cobb angle at baseline, the progression of the Cobb angle in 6 months of time was predicted. The correlation between the predicted and ground truth Cobb angles was calculated and presented in the Pearson correlation coefficient, and the coefficient is 0.86, as represented in Figure 9.

When performing Cobb angle progression prediction, the predicted Cobb angle was classified into five classes: Class A was the predicted Cobb angle smaller than 15 degrees, Class B was between 15 and 25 degrees, Class C was between 25 and 35 degrees, Class D was between 35 and 45 degrees, and Class E was larger than 45 degrees. The performance of the machine learning model in predicting the class of Cobb angle progression is summarized in Table 1. The overall accuracy of the model was 0.85, with a range of 0.72 to 0.99.

## 4. Discussion

### 4.1. Interpretation of the Findings

**Figure 10:**
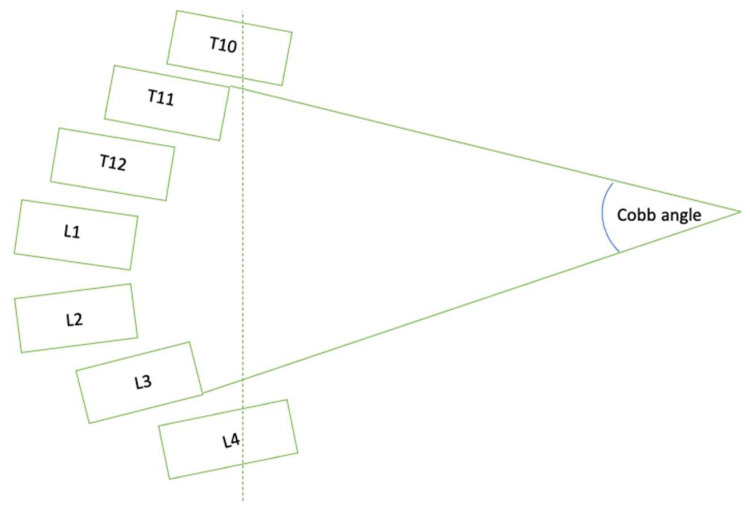
Schematic of Cobb angle measurement. In this case, the line drawn from the upper edge of the T11 vertebrae and the line drawn from the lower edge of the L3 vertebrae make up the Cobb angle.

According to the inter-vertebral angle-pair matrix (as shown in Figure 4), two pieces of information could be obtained—the largest inter-vertebral angle and its associated vertebral pair through extrapolation. The largest inter-vertebral pair angle extracted from the matrix was indeed the Cobb angle of the spinal curvature observed in the patient since the Cobb angle has been mathematically defined as the angle between the line extended from the upper end-plate of the vertebral body with the greatest inclination and the line extended from the lower end-plate of the vertebral body with the greatest inclination (which will be presented in Figure 10) [22].

Based on the magnitude of the spinal curvature (i.e., Cobb angle) and other relevant clinical factors such as the type of scoliosis and the number of remaining years of growth, doctors would be able to recommend the most appropriate option from a list of currently available treatments including continuous observation, brace wearing, and surgical interventions [23]. Since different treatments are specifically employed in a particular range of Cobb angles, another FNN that could classify the type of treatment the respective patients should receive was also created according to the inputted X-ray images. For instance, observation is only needed if the Cobb angle is less than 25 degrees, whereas wearing an orthotic brace is usually recommended for patients in the range of 25 to 50 degrees. If immature patients have Cobb angles greater than 45 degrees, surgical interventions will be needed [23]. Therefore, based upon the category that each individual patient has been assigned to, they shall receive the corresponding treatments.

Other than that, the multiple overlaps between the curves representing the ground-truth and predicted values of the spine curvature (as portrayed in Figure 5), which were observed in a majority of cases, indicated the high accuracy and precision of the deep learning model in terms of the prediction of the spinal curvature progression in the 6 months after the first clinical visit. Lastly, given that the relationship between the ground truth and predicted Cobb angle values is linear in nature, the strong positive correlation coefficient (i.e., +0.85) indicated a strong linear relationship between the two variables [24].

### 4.2. FNNs and Other Neural Networks

There are numerous advantages associated with the use of an FNN [25]. First, the neuronal nature of non-linearity; since the input and output are non-linear in their relationships, the non-linear nature of the neurons that form the neural network is an essential property for output data to be obtained from input data. Second, neuronal adaptability is another property possessed by an FNN, as neurons in an artificial neural network (ANN) can adapt to a dataset in the absence of the user. Third, there is noise resistance, which is supported by the fact that the performance of the FNN is invertedly associated with the noise level, which is opposite to the Partial Least Squares (PLS) neural network. At last, there is the map connection between the input and output. The network is initially presented to sets of examples where each consists of input data and their respective desired outcome for training purposes. During the training period, the weight coefficients between the neurons are changed in a such way that the differences between the predicted values and growth truth values are kept at the minimal [25].

As mentioned, an FNN, which fell into the category of an artificial neural network, was utilized for the calculation and prediction of Cobb angles from X-ray images. However, there are other existing neural networks that have been used for the same purpose, i.e., calculation and prediction of scoliosis progression. In the literature, a novel W-transformer network that was mainly composed of a hybrid transformer, reinforcement branch, and enhanced prediction module was employed for the automatic measurement of Cobb angles from X-ray images. Initially, local context information was extracted by feature extractors within a 10-layered convolutional neural network and was ensued by the extraction of global context information by a hybrid transformer. The reinforcement branch served as an intermediatory to improve the feature maps made by the feature extractor and transformer by lowering the number of landmarks that are either missing or redundant. The prediction module involved the prediction of key-points in the heatmap and offset map. Five heatmaps for five different keypoints in each vertebra were predicted for the localization of the landmark. To compensate for the loss of spatial information during the down-sampling procedure, “offset” was used to re-locate the keypoints back to their coordinates (in the original image) before the Cobb angle was calculated. As suggested by the paired *t*-test, there was no significant difference between the growth truth values (i.e., the Cobb angle manually calculated by experts) and the predicted Cobb angle values calculated by their model. Apart from that, four assessed parameters (accuracy, precision, sensitivity, and specificity) in the diagnosis of scoliosis (Cobb angle > 10 degrees) were, on average, greater than 80% [26].

In another instance, two deep learning algorithms, namely, faster-RCNN and RasNet, were employed for the localization of the patients’ backs from back photos and binary classification for the severity of the spinal curvature, respectively. The two algorithms involved in this approach were shown to have a high accuracy, sensitivity, and specificity (all, on average, greater than 90%) when it came to categorization into four classes of Cobb angles (i.e., <10 degrees, 10–19 degrees, 20–44 degrees, and greater than 45 degrees). To identify the back regions that were mainly linked to the spinal curvature of the individual patients, heatmaps were employed as additional measurements. It was revealed that the lumbar and scapula regions were mostly responsible for the clinical phenotype in the study cohort. Heatmaps were used to search for observable clinical phenotypes, which indicated the possibility of scoliosis, allowing potential patients to be screened radiographically and receive treatment at the earliest time possible to prevent further progression [7]

### 4.3. Improvements

Although the FNN rendered desirable results, some changes in the neural network will be needed for further improvements. The modification of the network architecture, the customization of loss functions, and ways to prevent over-fitting can all contribute to the enhancement of network performance, particularly in the field of pattern recognition [27,28]. To elaborate upon the matter of “over-fitting”, it can be minimalized in two ways: 1 “dropout” which essentially involves the random omission of hidden neurons with a probability of 0.5 of preventing their reliance and co-adaptations with other hidden neurons when each training set is being processed by the network. However, such action did not cause significant effects on the learning process of the network [7,27,28]. When 50% “dropout” was implemented upon all the hidden neurons, the amount of test error was significantly reduced from 160 errors to 130 errors. Similarly, upon the implementation of the 50% “dropout” strategy on all hidden Markov models (HMMs) in speech recognition systems with different network architectures, the speed of recognition was enhanced by approximately 3% [29]. The second approach is the use of regularization strategies that are introduced for the reduction in network complexity through imposing penalty terms that set constraints upon the growth of weights and reduce the size of errors during the training period. It is also important in maintaining a balance between the amount of available information in the training dataset and the network complexity to achieve a desirable generalization [30]. Penalty terms can be categorized into L1 and L2 penalty terms, and they are represented as lambda (_λ_) within the cost function formula, where an L1 penalty reduces the absolute value of the weights and L2 reduces the squared value of the weights [30]. Since the penalty forms are intended to prevent weights from growing in the normal circumstances (“weight decay”), which will reduce their impacts on the learning process, the network will be more focused on features that appear at higher frequencies in the training set, which will better enhance generalization [31,32].

### 4.4. Further Investigations

Since the study did not include all the parameters as inputs for the training and testing of the network, additional parameters such as Risser’s sign and gender can be introduced as inputs to the FNN in order to investigate if they have potential impacts on the existing weights between neurons and, thus, the performance of the network. Changes in the network architecture are also one of the considerations for future studies. The FNN can be modified in the following respects: the number of hidden layers, the number of neurons in each layer, and the incorporation of unique properties from other neural networks in order to enhance its predictive capabilities. Assessment parameters (e.g., accuracy rate, misclassification rate) derived from the confusion matrix can be used to investigate if the performance of the network has been enhanced.

Considering the fact that the FNN was not compared to other neural networks in this study when it came to their respective predictive capabilities in scoliosis progression, a comparative study could be conducted such that the essential properties, e.g., the architecture of the network, could be determined to build the network with the ability to make the most accurate and reliable prediction regarding the scoliosis progression.

## 5. Conclusions

To conclude, the largest inter-vertebral angle pair derived from the above matrix and the predicted Cobb angles in 6 months of follow-up calculated by the FNN rendered one of the most meaningful pieces of clinical information: the range of Cobb angles patients would fall into and the corresponding treatments that could be prescribed to prevent the progression of scoliosis. It was shown that an FNN was able to make predictions on scoliosis progression with a high accuracy, sensitivity, and specificity. However, “over-fitting” might be one of the many problems that contributed to the unsatisfactory performance of the FNN, and it could be addressed by either the 50% dropout or the L1 and L2 regularization strategies through the prevention of the co-adaptation of the neurons and “weight-decay” respectively.

## Figures and Tables

**Table 1:** Predicted Cobb angles classifications, accuracy, sensitivity, and specificity.

	Class	Accuracy	Sensitivity	Specificity
Predicted Cobb Angle	Class A: <15°	0.84	0.78	0.86
	Class B: >15° <25°	0.72	0.65	0.76
	Class C:>25° < 35°	0.80	0.60	0.88
	Class D: >35° < 45°	0.94	0.28	0.99
	Class E: >45°	0.99	0.99	1.00
	Overall	0.85	0.65	0.91

## Data Availability

The datasets used and/or analyzed during the current study are available from the corresponding author on reasonable request.

## Abbreviations

3D: three-dimensional; AI: Artificial Intelligence; AIS: adolescent idiopathic scoliosis; ANN: artificial neural network; FNN: Feedforward Neural Network; HMMs: hidden Markov models; PA: posteroanterior; PLS: Partial Least Squares; QUS: quantitative ultrasound; ROI: region of interest; SMAPE: symmetric mean absolute percentage error.
